# Supplementary material for: Label-free spatio-temporal monitoring of cytosolic mass, osmolarity, and volume in living cells
Source: Nat Commun. 2019 Jan 21;10:340. doi: 10.1038/s41467-018-08207-5 (PMC6341078; doi:10.1038/s41467-018-08207-5)
Supplement: Supplementary file 1 — Supplementary Information [file 41467_2018_8207_MOESM1_ESM.pdf]

Supplementary information

Label-free, spatio-temporal monitoring of cytosolic mass, osmolarity and volume, in living cells

Midtvedt et al.

Supplementary Note 1

Optical setup

A sketch of the setup employed is shown in Supplementary Figure 1A. The laser beam is first expanded and passed through a half-wave plate (marked with arrows in the figure), and is subsequently split into two paths by a polarizing beam splitter (marked by PBS). The latter two components provide control of the light polarization as well as the relative light intensity in the reference and object arm. A second half wave plate is implemented in the object beam to ensure aligned polarizations of the object and reference beam. The object beam is passed through the sample, as well as a microscope objective (marked MO) and a tube lens (marked TL). The two beams are recombined using another beam splitter (marked BS), which is slightly tilted to ensure the off-axis configuration. The intensity of the recombined field is then measured by a camera (marked C). A millifluidic chip (Supplementary Figure 1B) provides controlled solution handling.

Hologram reconstruction

Off-axis digital holography relies on the numerical reconstruction of the optical field that is scattered off an object from a recorded hologram. Physically, the hologram is the interference pattern formed by the scattered optical field and a reference beam which does not pass through the sample. The intensity recorded by the camera is given by

$$I_{cam} = \left|E_{sca} + E_{ref}\right|^2 = |E_{sca}|^2 + \left|E_{ref}\right|^2 + 2|E_{sca}| \cdot \left|E_{ref}\right| \cos(\boldsymbol{k_p} \cdot \boldsymbol{x} + \phi(x,y)) \quad (1)$$

where E_{sca} is the scattered field, E_{ref} is the reference field, $\boldsymbol{k_p}$ is the projection of the wavevector of the reference signal on the camera plane $\boldsymbol{x} = (x, y)$ and $\phi(x,y)$ is the phase of the scattered field (Supplementary Figure 1C). In Fourier-space, the last term in the expression above is split into two peaks at $\boldsymbol{k} = \pm\boldsymbol{k_p}$ (see Supplementary Figure 1D). Assuming a uniform intensity of the reference field, this enables reconstructing the scattered field (and its phase, Supplementary Figure 1E) by multiplying the measured intensity by a complex phase factor $\exp(-i\boldsymbol{k_p} \cdot \boldsymbol{x})$ and applying a low-pass filter to this modified intensity. In our measurements, we used a low pass-filter with cutoff-wavenumber $k_{max} = 0.4|\boldsymbol{k_p}|$. Since this cutoff sets a limit to the obtainable resolution, we aimed at choosing the off-axis angle such that $|\boldsymbol{k_p}|$ was maximized without violating the Nyquist sampling theorem. The pixel size of our setup ($dx = 0.08$μm) restricts the off-axis angle to $\left|\boldsymbol{k_p}\right| < \frac{2\pi}{4dx} = \frac{\pi}{0.16}\,\mu\mathrm{m}^{-1}$. We found that an off-axis angle corresponding to $\frac{2\pi}{6dx} > \left|\boldsymbol{k_p}\right| > \frac{2\pi}{8dx}$ provided stable phase images without interference from the zero order intensity while being sufficiently far away from the Nyquist limit.

We found it necessary to post-correct the phase maps for spherical distortions in the field detected by the camera. In order to avoid 2π-ambiguities in the fitting procedure, we constructed the gradient of the phase map as $\Delta\phi_\lambda^{i,j} = -\pi + \mathrm{mod}(\pi + \delta\phi_\lambda^{i,j}, 2\pi)$, where $\lambda = (x, y)$ and $\delta\phi_\lambda^{i,j}$ is the discrete differential operator at pixel index i, j in direction λ. The phase gradient was then fitted, using linear least squares, to the gradient of a parabola $\hat{\phi}(x,y) = A\,x^2 + By^2 + Cxy + Dx + Ey$. This function was then subtracted from the experimentally obtained phase map to correct for distortions in the field.

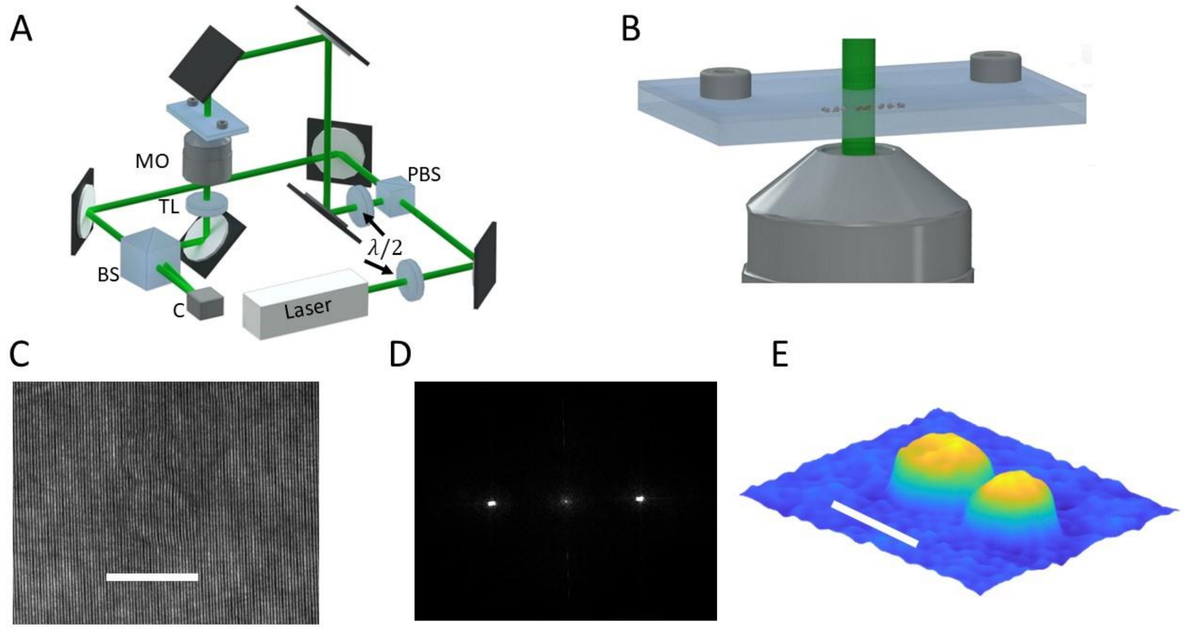

Supplementary figure 1 (A) Sketch of the optical setup used in the study. (B) Cells are immobilized on the floor of a millifluidic chip which allows for controlled solution exchange. (C) The interference pattern recorded by the camera. Scale bar is $10\mu m$. Note that two yeast cells are vaguely visible in the center of the image. This image represents only a fraction of the full field of view of the camera, which is $154\mu m$ by $115\mu m$. (D) In Fourier space, the interference pattern is split into three distinct peaks. Each of the off-center peaks contains identical information about the phase shift of the light passing through the sample. (E) Quasi three dimensional rendering of the phase map of the two cells seen in figure 1C. Scale bar is $10\mu m$.

Supplementary Note 2

Cell segmentation

In order to extract single-cell data from a cluster of yeast cells, such as the one shown in figure 1, it is crucial to robustly estimate the cell borders to provide accurate cell segmentation. In order to avoid introducing subjective measures for determining cell borders (such as manual thresholding of images) we aim at defining a basis function $\Psi(x, y; \boldsymbol{p})$ which can be projected onto the experimentally measured phase shift of a cell. Such a basis function should reproduce important features of the phase response, and take a set of parameters $\boldsymbol{p}$ defining the physical properties of the cell. To avoid overfitting, we also aim at making the set of cell parameters as small as possible. The simplest conceivable basis function takes only two parameters as input; namely cell size and the magnitude of the phase shift. Further, we found that an additional parameter related to the resolution of the microscope is needed in order to reproduce the phase behavior of yeast cells of varying sizes.

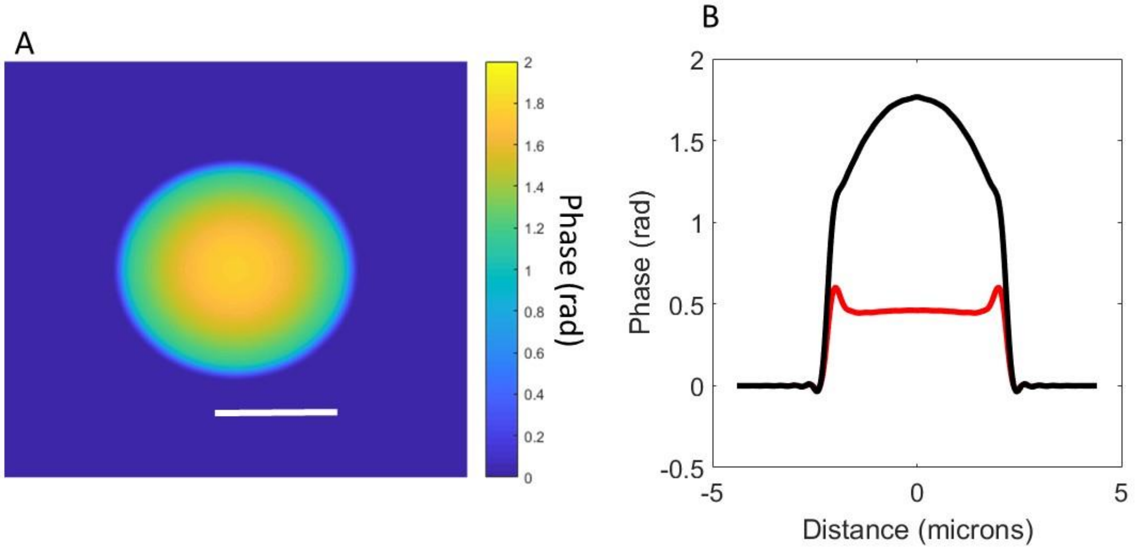

Supplementary figure 2: (A) Simulated phase distribution of a spherical model cell having cytoplasmic RI of 1.37 and a cell wall RI of 1.41. The cell wall thickness was taken to be 200 nm. The cell environment has an RI of 1.33. Scale bar is 2 µm. (B) Line scan of the phase shift through a cell with parameters as in (A) but in different environments (Black line: medium RI of 1.33, red line: medium RI of 1.363).

In order to develop such a basis function, we performed numerical simulations based on the beam propagation method to estimate the expected phase shift from yeast cells. The yeast cells were modeled as being composed of a spherical cytoplasm with radius r_{cyt} and homogeneous refractive index (RI) $n_{cyt} = 1.37$, encapsulated inside the cell wall having a thickness d_{wall} and RI $n_{wall} = 1.41$ (see Supplementary Figure 2A). The basis function should provide a reliable measure for the cell phase shift under a large variety of conditions and cell sizes. We therefore systematically studied the shape of the phase shift for varying cell sizes, varying cell wall thicknesses and varying extracellular RI. We found that the shape of the expected phase response changes dramatically when the extracellular RI is close to the cytoplasmic RI, in which case the phase signal is dominated by the cell wall (figure 2B). In order to accommodate this observation, we explicitly avoid including any assumptions on the shape of the phase shift in the basis function. On the basis of these simulations we chose the following basis function,

$$\Psi(\rho; R, \phi_0, \sigma) = \frac{\phi_0}{2}\left(1 + \tanh\left(\frac{R-\rho}{\sigma}\right)\right) \quad (2)$$

This function predicts a flat phase response for the radial coordinate $\rho < R$ and an exponential decay with characteristic length σ. The integrated phase response of individual cells is then obtained as,

$$\Phi(R, \phi_0, \sigma) = 2\pi \int_0^\infty d\rho\, \rho\, \Psi(\rho; R, \phi_0, \sigma) \approx \pi\phi_0 R^2 \quad (3)$$

In order to estimate the effects on refraction at the cell interface, we performed a numerical investigation of the integrated phase shift defined by Supplementary Eq. 3 for cells of varying radius (with fixed cytoplasmic RI=1.37) and cytoplasmic RI (with fixed cell radius=2 µm) (Supplementary Figure 3). We found excellent agreement between Supplementary Eq. 3 and the expected phase response when ignoring refraction (see Eq. 2 in main text) indicating that light refraction at the cell interface is indeed negligible in our case.

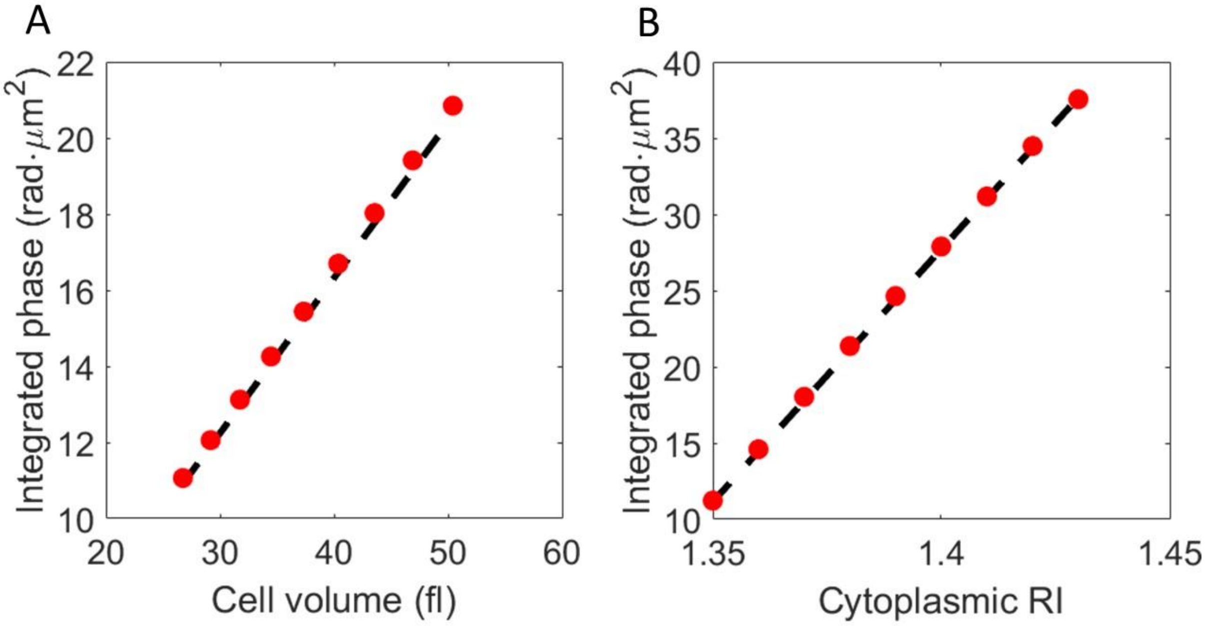

Supplementary figure 3: Scaling of the integrated phase response of individual cells of varying radius (A) and cytoplasmic RI (B) from BPM simulations (red circles) and the theoretical expectation based on negligible light refraction (dashed lines). In these simulations the cell wall was assumed to have RI=1.41 and occupy 30% of the total cell volume.

The parameter R roughly represents the distance from the cell center at which the phase shift is half its maximum value. For a spherical cell with radius R_{cell} and homogeneous RI, one has $R = 6/7R_{cell}$. Although the presence of a cell wall with a slightly different RI than the cytoplasm shifts this value slightly, we have opted for using the value $6/7$ for the proportionality constant in the presentation of our results. However, since all our measurements rely only on the integrated phase response defined by Supplementary Eq. 3 and relative changes in projected cell area, the exact value for the proportionality constant does not influence the results.

This basis function allows to quantify single-cell responses in cell clusters containing cells of varying size and morphology. To achieve this, each phase map is projected onto a set of such basis functions by minimizing the following expression,

$$F = \sum_{i,j}\left(\phi_{i,j} - \sum_{k=1}^{N} \Psi(\sqrt{(i-i_k)^2 + (j-j_k)^2}; R_k, \phi_{0k}, \sigma_k)\right)^2 \quad (4)$$

where $\phi_{i,j}$ is the phase shift measured at pixel i, j, N is the number of cells in the phase map, $i_k, j_k, R_k, \phi_{0k}, \sigma_k$ are the midpoint location, radius, phase shift and characteristic phase decay length of cell number k. To exemplify the implementation of this method, we show in figure 4 the segmentation of 11 cells using this basis function.

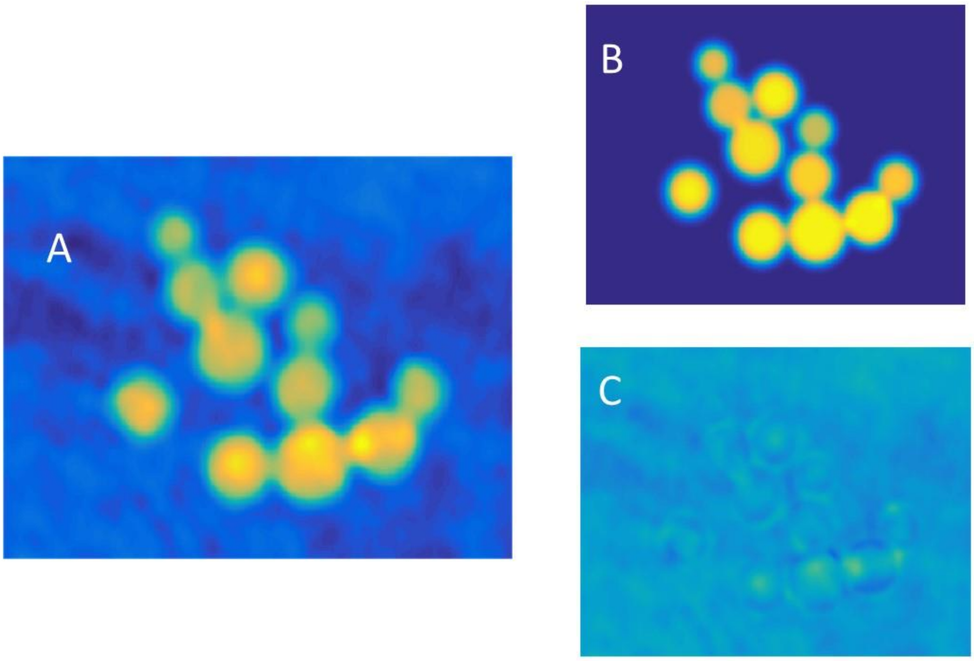

Supplementary figure 4: Procedure of cell segmentation. A: The original image of the cell cluster. B: Projected phase image of the cell cluster using the basis function defined in the text. C: Result after segmentation. All cells successfully segmented.

Supplementary Note 3

Noise estimation

The ability of our setup to resolve small spatiotemporal changes in cell mass distribution is limited by the phase noise of the system. This noise partially results from the pixelation and bit depth of the camera, but mainly reflects the spatiotemporal fluctuations in the relative path lengths of the object and reference beam, primarily due to roughness and dust on glass surfaces in the beam path.

In order to quantify the performance of our system, we first treat theoretically the response in the reconstructed field due to small perturbations in the intensity detected by the camera. For simplicity, we will treat the holographic imaging of a one-dimensional phase object; the extension to a two-dimensional system is straightforward but algebraically inconvenient. The intensity at the camera plane is written as,

$$I(x) = I_0 + I_1 \cos\big(k_0 x - \phi(x)\big) \tag{5}$$

where I_0 is the background intensity formed by the two beams, k_0 is the wavevector quantifying the off-axis angle, $\phi(x)$ is the phase of the object and I_1 is the amplitude of the interference pattern, given by $2|E_{ref}||E_{obj}|$ where E_{ref} is the reference field and E_{obj} is the field transmitted through the specimen. This field can be reconstructed as,

$$E_{obj}(x) = \frac{1}{2} k_m |E_{ref}|^{-1} \int dx' \mathrm{sinc}(k_m(x - x'))\, I(x') \mathrm{e}^{-ik_0 x'} \tag{6}$$

where k_m is a cutoff wavelength. For mathematical convenience we model the intensity perturbation as a Gaussian white-noise component added to the measured intensity; $I(x) \to I(x) + \delta I(x)$, with $\langle \delta I(x) \rangle = 0$ and $\langle \delta I(x) \delta I(x') \rangle = S^2 \delta(x - x')$. Similarly, the reconstructed field is augmented by a noise term as well, given by,

$$\delta E(x) = \frac{1}{2} k_m |E_{ref}|^{-1} \int dx' \mathrm{sinc}(k_m(x - x'))\, \delta I(x') \mathrm{e}^{-ik_0 x'} \tag{7}$$

Its autocorrelation is thus given by,

$$\langle \delta E(x)\delta E(x')^*\rangle = \frac{1}{4}k_m^2\left|E_{ref}\right|^{-2}\int dx''\int dx'''\mathrm{sinc}\big(k_m(x-x'')\big)\mathrm{sinc}\big(k_m(x'-x''')\big)\langle \delta I(x'')\delta I(x''')\rangle \mathrm{e}^{-ik_0(x''-x''')} \quad (8)$$

which can be explicitly evaluated using $\langle \delta I(x)\delta I(x')\rangle = S^2\delta(x-x')$ to,

$$\langle \delta E(0)\delta E(x)^*\rangle = \frac{1}{4}k_m^2 S^2\left|E_{ref}\right|^{-2}\int dx''\mathrm{sinc}(k_m x'')\mathrm{sinc}\big(k_m(x-x'')\big) = \frac{1}{4}k_m S^2\left|E_{ref}\right|^{-2}\mathrm{sinc}(k_m x). \quad (9)$$

where we have utilized the fact that the autocorrelation depends only on the lag which allows us to make the replacement $x'-x \to x$. Thus, as a result of the filtering of the signal in Fourier space an uncorrelated input noise is transformed into a correlated noise in the reconstructed field, with a correlation length that depends on the size of the filtering window in Fourier space.

We next investigate how this correlated noise in the reconstructed field affects the autocorrelation of the reconstructed phase. We assume a flat phase map, and arbitrarily set $\phi(x) = 0$ everywhere. The transmitted field E is thus completely specified by its real valued amplitude E_0. Due to the noise term, this field is augmented by a complex field $\delta E(x)$ with the correlation properties specified above, so the reconstructed field becomes $E_{obj} = E_0 + \delta E(x)$. Since there is no preferred phase of this added field, its real and imaginary parts will be identically distributed independent random variables, so that

$$\langle \mathrm{Im}\{\delta E(0)\}\mathrm{Im}\{\delta E(x)\}\rangle = \langle \mathrm{Re}\{\delta E(0)\}\mathrm{Re}\{\delta E(x)\}\rangle = \frac{1}{8}k_m S^2\left|E_{ref}\right|^{-2}\mathrm{sinc}(k_m x). \quad (10)$$

The phase of the reconstructed field is given by $\phi_{obj}(x) = \mathrm{atan}\left(\frac{\mathrm{Im}\{\delta E(x)\}}{E_0+\mathrm{Re}\{\delta E(x)\}}\right) \approx \frac{\mathrm{Im}\{\delta E(x)\}}{E_0}$.

The autocorrelation in the reconstructed phase thus becomes,

$$\langle \phi_{obj}(0)\phi_{obj}(x)\rangle = \frac{1}{2}k_m\left(\frac{S}{I_1}\right)^2\mathrm{sinc}(k_m x), \quad (11)$$

where I_1 is the amplitude of the interference pattern. Note that in the limit $k_m \to \infty$ this reduces to $\langle \phi_{obj}(0)\phi_{obj}(x)\rangle = \frac{1}{2}\left(\frac{S}{I_1}\right)^2\delta(x)$. This result can be generalized to $\langle \left|\phi_{obj}(\boldsymbol{k})\right|^2\rangle = \frac{1}{2}\left(\frac{S}{I_1}\right)^2 F_{\boldsymbol{k}_m}(\boldsymbol{k})$ where $F_S(\boldsymbol{k})$ is the shape, in Fourier space, of the low-pass filter employed in the reconstruction of the field. From this we infer that to maximize the phase resolution, the amplitude I_1 should be as large as possible. Taking the intensity fluctuations to result solely from the bit-depth of the camera, we estimate the bit-depth limited phase resolution to $\langle \phi_{limit}^2\rangle = 2^{-(2b-1)}$ where b is the bit-depth. In our case, using an 8-bit camera, this limits the phase resolution to $\langle \phi^2\rangle > 2^{-15}\mathrm{rad}^2$. In figure 5 we show the measured autocorrelation of the phase noise of our system together with the theoretically expected phase noise, with the amplitude of the intensity noise as the only input parameter. The measured phase noise is well described by the theoretical considerations presented above, with noise level $k_m\left(\frac{S}{I_1}\right) \approx 0.22$.

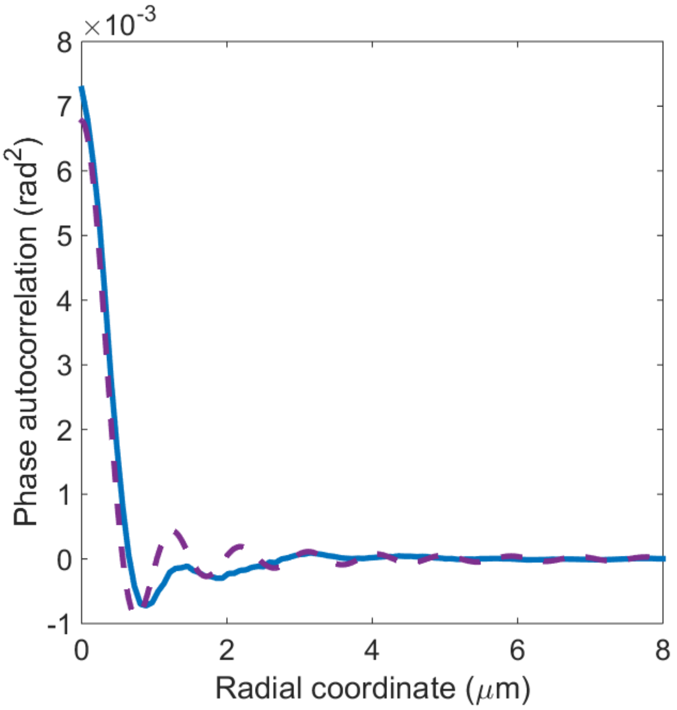

Supplementary figure 5: Autocorrelation of phase noise. The full line is the measured phase noise while the dashed line is the theoretically expected noise correlation.

Next, we investigate how the correlations in the phase noise limits the determination of the integrated phase shift, and thus the cell mass. The integral Φ_S of the phase shift over a region S is itself a stochastic variable, with variance $\mathrm{Var}[\Phi_S] = \int_S dx \int_S dx' \langle \delta\phi(x)\delta\phi(x')\rangle$. Denoting the autocorrelation of the phase noise as $C(|x-x'|) \equiv \langle \delta\phi(x)\delta\phi(x')\rangle$ we use the Wiener-Khinchin theorem and the Plancherel theorem to rewrite this expression as $\mathrm{Var}[\Phi_S] = \int dk\, |F_S(k)|^2 \hat{C}(k)$ where $\hat{C}(k)$ is the Fourier transform of the autocorrelation function and $F_S(k)$ is the Fourier transform of the integration region. In the one-dimensional case considered above, taking the region S to be the interval $x \in [-a, a]$, this amounts to,

$$\mathrm{Var}[\Phi_S] = \frac{1}{2}\left(\frac{S}{I_1}\right)^2 \int dk\, |F_S(k)|^2 F_{k_m}(k) =$$
$$2a^2\left(\frac{S}{I_1}\right)^2 \int_{-k_m}^{k_m} \mathrm{sinc}^2(ka)dk = (2\pi)^{-1}\, k_m^{-1}\left(\frac{S}{I_1}\right)^2 \left(\cos 2ak_m - 1 + 2ak_m \mathrm{Si}(2ak_m)\right) \equiv$$
$\frac{a}{2}\left(\frac{S}{I_1}\right)^2 f(ak_m)$. The function $f(x) \to 1$ as $x \to \infty$ as expected when considering the limit $k_m \to \infty$.

Thus, when estimating the integrated phase of large objects (such that $ak_m \gg 1$) the correlations in the phase noise can be neglected and the variance of the integral scales linearly with the size of the region. In contrast, for $ak_m \sim 1$ the details of the correlations has to be taken into account to correctly estimate the influence of the phase noise.

In our system, the cutoff wavenumber is typically taken to be approximately $4 - 6\mu\mathrm{m}^{-1}$, suggesting that when studying objects smaller than approximately 500nm the phase noise correlations needs to be taken into account. The objects of interest in this study are larger ($1 - 2\mu\mathrm{m}$), and thus the variance of the integrated phase scales with cell area A as $\mathrm{Var}[\Phi_S] \sim A\left(\frac{S}{I_1}\right)^2$. The relative error in the mass determination is then estimated as $\frac{\delta m}{m} = \frac{\sqrt{\mathrm{Var}[\Phi_S]}}{\Phi_S} \sim R^{-2}\left(\frac{S}{I_1}\right)$ where R is the cell radius and where we have assumed that the object is spherical so that $\Phi_S = 4\pi k \frac{R^3}{3}\, \Delta n$.

However, holographic imaging is often used to characterize smaller objects as well, such as bacteria and nanoparticles. Due to the phase correlations the relative error in the mass determination of smaller objects does not follow the same scaling. Evaluating the variance of the integrated phase noise explicitly in the two-dimensional case we find,

$$\mathrm{Var}[\Phi_S] = \pi R^2 \left(\frac{S}{I_1}\right)^2 \left(1 - J_0(k_m R)^2 - J_1(k_m R)^2\right) \tag{13}$$

where $J_\nu(x)$ is the Bessel function of order ν. For small arguments, $k_m R < 4$, this is well approximated by $\mathrm{Var}[\Phi_S] = \left(\frac{S}{I_1}\right)^2 \frac{\pi R^2 (k_m R)^2}{8+(k_m R)^2+(1/18)(k_m R)^4}$. Requiring that $\mathrm{Var}[\Phi_S] < 0.25\Phi_S^2$ for detection of a particle, and writing $\Phi_S = 4\pi k \frac{R^3}{3} \Delta n$ for a spherical nanoparticle, we find that the limit of detection of our system depends on the particle size and refractive index as $R\Delta n \geq \frac{3}{4}\left(\frac{S}{I_1}\right) \left(\frac{k_m}{k}\right) (2\pi)^{-1/2}$. Using the noise level measured above this amounts to $R\Delta n \geq 7\mathrm{nm}$. Thus, our setup is sensitive to local changes in optical path length $\geq 7\mathrm{nm}$.
